# Factors Influencing the Survival Rate of Teeth and Implants in Patients after Tumor Therapy to the Head and Neck Region—Part 2: Implant Survival

**DOI:** 10.3390/jcm11216319

**Published:** 2022-10-26

**Authors:** Ramona Schweyen, Waldemar Reich, Peter Jevnikar, Thomas Kuhnt, Andreas Wienke, Jeremias Hey

**Affiliations:** 1 Department of Prosthetic Dentistry, University School of Dental Medicine, Martin Luther University Halle-Wittenberg, 06112 Halle, Germany; 2 Department of Oral and Maxillofacial Plastic Surgery, Martin Luther University Halle-Wittenberg, 06120 Halle, Germany; 3 Department of Prosthodontics, Faculty of Medicine, University of Ljubljana, SI-1000 Ljubljana, Slovenia; 4 Department of Radiotherapy, University Clinic, University Leipzig, 04103 Leipzig, Germany; 5 Department of Medical Epidemiology, Biometry and Computer Science, Martin Luther University Halle-Wittenberg, 06112 Halle, Germany

**Keywords:** head and neck cancer, prosthetic rehabilitation, implants, risk factors, radiation therapy

## Abstract

During prosthetic rehabilitation after tumor therapy (TT) in the head and neck region, the dentist must assess whether the prognosis of the remaining teeth is sufficiently good or whether implants should be used to anchor dentures. Thus, the aim of the present study was to compare the survival rate of teeth and implants after TT and to evaluate factors potentially influencing implant survival. One hundred fifteen patients (male: 70.3%; mean age: 63.2 ± 12.4 years) having received dental treatment before and after TT at the Martin Luther University Halle-Wittenberg were enrolled in the study. Clinical examination including assessment of dental status and stimulated salivary flow rate was performed. Information about disease progression and therapy was retrieved from medical records. After TT, from a total of 1262 teeth, 27.2% had to be extracted. Of 308 implants inserted after TT, 7.0% were lost. Teeth exhibited lower 5-year survival probability (76.8%) than implants (89.9%; *p* = 0.001). The risk of loss (RL) of implants increased with age, nicotine use, intraoral defects, and RCT. Radiotherapy did not independently increase the RL. Thus, implants seem to be a reliable treatment option in case of progressive tooth decay after TT, particularly after RT.

## 1. Introduction

After tumor therapy (TT) to the head and neck region, the treating dentist must consider a variety of posttherapeutic side effects among patients. Besides resection-related anatomical changes, restrictions in tongue and mandibular mobility, and difficulties in mouth opening, xerostomia is considered the most serious sequela, especially in irradiated patients [1,2,3]. Often, oral hygiene becomes more challenging, increasing susceptibility to caries [4,5]. The treatment of radiation caries is often extremely frustrating for both the dentist and the patient because it cannot be stopped despite close monitoring and prompt conservative measures. Therefore, in many cases, it is necessary to first remove a few teeth and then, in severe cases, all remaining teeth [4,6].

There are currently no guidelines regarding the design and type of dental prosthesis after TT to the head and neck region. Therefore, the therapeutic decision for the rehabilitation of patients after TT is discretionary. It is the dentist’s task to decide whether the prognosis of the remaining teeth is good enough to be used as abutments for dentures. If the prognosis of the teeth is unfavorable or patients cannot adapt to a removable prosthesis due to anatomical and/or functional changes, the placement of endosseous implants is recommended earliest after one year of freedom from recurrence [7].

In principle, the same criteria for successful implantation are applied as defined in the literature for implantation in patients without tumor history [8]. Implant success is described as “ideal clinical conditions over a period of at least 12 months for implants serving as prosthetic abutments.” To achieve such a result, it is essential to consider a variety of factors: healthy bony and mucosal tissue conditions in the area of the future implant site, presurgical evaluation of bone density, precise measurement of the bone volume to determine the ideal implant length and diameter, selection of the correct implant type with adequate surface treatment, experienced execution of the implant placement to achieve sufficient primary stability, adequate soft-tissue management during and after exposure, a well-thought-out design of the prosthetic restoration, and the patient’s motivation to comply with the necessary prophylactic measures during the follow-up care [9,10,11].

However, the conditions desirable for implantation are rarely found in patients with a condition following TT in the head and neck region. After radiotherapy (RT), osseointegration is thought to be compromised because of the decreased healing and remodeling potential of bone tissue due to fibrosis and the lack of vascularization [12]. Diminished blood supply to the mucosa and hyposalivation cause periodontal and peri-implant inflammations [7]. Immunological defense mechanisms function only to a limited extent, and every surgical intervention, especially in the bone, carries the risk of infection. To avoid the most serious side effect after RT, osteoradionecrosis (ORN), elective implant insertion was critically evaluated for a long time [12]. However, if conventional mucosal-supported dentures cannot be worn due to anatomical or functional changes, implants are often the only way to successfully anchor dentures. If radiation doses are taken into account, previous studies have proven the success of implant insertion [12,13].

The benefit of implants must be carefully considered against the risk of damage to the vulnerable mucosa and the ORN risk to the bone. Concerning implant survival, the current literature has discussed various causal factors. In addition to age, sex, salivary flow rate (SFR), and history of stimulant use, which have a certain influence on implant survival in patients without tumor history, the influence of the implant site (autologous bone vs. grafts) and any additional RT and RT with chemotherapy (RCT) that may have taken place are discussed as important factors in patients with a condition after TT in the head and neck region [13,14,15]. Systematic studies comparing the survival of teeth and implants are not yet available.

In Part 1 of our study, we evaluated the factors potentially influencing the survival of teeth [16]. The present study aimed to compare the survival of teeth and implants after TT to the head and neck region in the same cohort. It was postulated that implants exhibit a higher probability of survival after TT than the patients’ own teeth. In addition, we evaluated possible factors influencing implant survival, such as age, sex, SFR, nicotine consumption, implant site, and RT or RCT.

## 2. Materials and Methods

One hundred fifty-three patients who underwent TT due to head and neck cancer between 1985 and 2018, including those undergoing dental follow-up after completion of TT and oral rehabilitation from 1 January 2019 to 31 January 2020 at the University Clinic for Prosthodontics, were initially included in the present study. Thereof, 35 patients with advanced age- or disease-related general health or cognitive impairment, those with premature death, and those not provided informed consent were excluded. The study protocol was approved by the Ethics Committee of the Medical Faculty of Martin Luther University Halle-Wittenberg (Nos. 2017-62 and 2018-130). The study’s design was previously described in Part 1 of our manuscript [16]. For this reason, we briefly describe the study design below.

### 2.1. Tumor Therapy

The type of TT was determined and conducted by the treating clinics, primarily oral and maxillofacial surgery, otorhinolaryngology, and radiotherapy at the University Hospital Halle. The teeth in direct relation to the tumor or the area of the safety distance, as well as the teeth that could not be preserved because of decay, were generally removed during tumor-removal surgery.

### 2.2. Dental Treatment in Relation to Radiotherapy

Before RT, all patients were referred to the Department of Dentistry, Oral, and Maxillofacial Surgery by the University Clinic for Radiotherapy. The focal dental treatment performed here was based on the recommendations of the German Society for Dental, Oral, and Maxillofacial Medicine and is extensively described in previous publications [17,18,19,20,21]. Instructions on the handling of the radiation protection splints, oral hygiene, and nutrition during and after RT were provided to the patients in the form of brochures [22].

### 2.3. Dental Treatment after TT

All patients were recommended to follow a frequent quarterly follow-up regimen. This included a dental checkup, professional tooth cleaning, and timely treatment of any defects in the dental hard tissues. Carious lesions were treated following the recommendations of Kielbassa et al. [23] and Grötz et al. [24]. In the case of progressive generalized decay, the aim was to crown the remaining teeth as soon as possible.

In cases where adequate retention of a conventional prosthesis was not achievable, the possibility of implant placement was considered. Implant-prosthetic rehabilitation was planned during a joint consultation session by an experienced dental staff member of the University Clinic of Prosthodontics and an experienced dental or medical staff member of the University Clinic of Oral and Maxillofacial Surgery after one year of freedom from recurrence. In the case of edentulous jaws, four implants were usually planned for the anchorage of removable partial dentures; in particularly favorable conditions (sufficient mouth opening, well-preserved alveolar bone, and stable soft-tissue conditions), six implants were also placed. In the case of a condition after RT, the course of the isodoses in the area of the planned implant positions was evaluated beforehand. For isodoses exceeding 50 Gy, implant placement was not advised due to increased risk of ORN but was not denied [25].

If bone augmentation was required, only autologous bone material was used. The implantation itself was template-oriented, and the aim was to achieve closed healing. Implants from Straumann GmbH (Freiburg, Germany) and Astra Tech (Dentsply Sirona, Charlotte, NC, USA) were used. The healing time was at least four months.

If the implants were easily accessible for oral hygiene, then stable peri-implant mucosal conditions, as well as sufficient mouth opening, were present, and bar constructions were fabricated on four healed implants. If the implants could be inserted in optimal positions, telescopes were fabricated as an alternative. In the case of considerably reduced mouth opening, single connection elements with a lower construction height, mostly locators (Zest Dental Solutions, Carlsbad, CA, USA), were used. In addition, magnets (steco-system-technik, Hamburg, Germany) were selected for anchoring the prosthesis in cases of manual tactile-restricted patients, difficult accessibility to the implants due to pronounced scarring and/or restricted mouth opening, and premature implant failure. In individual cases, fixed-bridge restorations were also fabricated with good accessibility and stable peri-implant soft-tissue conditions. Easy cleanability of the fixed restoration was particularly important.

### 2.4. Data Collection

The same examiner collected the data. Information on anamnesis, demographic data, course of disease, and TT, as well as information on implant survival after TT, were obtained from the medical records. The dental status was determined by clinical examination, the presence of intraoral defects checked, and the stimulated SFR assessed [24,25,26,27,28,29]. For detailed information, please refer to our first manuscript [16].

### 2.5. Statistical Analysis

Statistical analysis was performed with the support of the Institute of Epidemiology at Martin Luther University Halle-Wittenberg. First, the epidemiological characteristics of the study cohort were presented descriptively. The survival-time data analyses of the teeth and implants after TT and the evaluation of possible influencing factors were performed using the Kaplan–Meier method and log-rank test. Final multivariable analysis evaluation regarding the influence of age, sex, chemotherapy, SFR, implant position, defect situation, tumor location, and implant site on the implants’ survival was performed. The affiliation of multiple implants to a patient (clustering) was considered using the marginal model with calculation of hazard ratios and robust 95% confidence intervals (CIs) [30]. In the marginal model, the inclusion of clustering leads to an increase in standard errors compared with Cox regression. To improve the sharpness of the test, the variables “defect situation” and “implant site” were dichotomized, i.e., divided into “intraoral defect present” and “no intraoral defects” as well as “autogenous bone” and “grafted bone.” The duration of the dental survival analyses extended from the end date of TT to the date of extraction or the examination date. The duration of the implant survival analyses extended from the date of implant insertion to the date of implant removal or to the examination date. Microsoft Excel 2016 (Microsoft Deutschland GmbH, Munich, Germany) and IBM SPSS Statistics, version 25.0 (IBM Inc., Ehningen, Germany) were used for analysis.

## 3. Results

### 3.1. Characterization of the Study Cohort

Data from 118 patients were included in the study (male: 70.3%; mean age, males: 63.2 ± 12.4 years, females: 63.0 ± 13.4 years). Regular tobacco use was reported by 49.4% of men and 37.1% of women. Clinical examination of the patients was conducted at an average of 80 ± 67 months after the end of primary TT. Table 1 lists details about the tumor localization and stage.

In the 107 patients who underwent primary surgery, no intraoral reconstruction was required in 19 cases (17.6%), such as laryngeal/hypopharyngeal malignancies, and no reconstruction was performed in six cases (5.6%), such as hemi-maxillectomy. In the remaining 82 cases, the resection defects were either primarily closed or covered by local or microvascular anastomosed flaps. Primary-defect coverage was achieved in 39 cases (36.1%). In eight patients (6.8%), local flap plasty, such as nasolabial flaps, was used to cover the defect. This included one patient who had not undergone primary surgery but underwent resection of necrotic bone followed by defect coverage using nasolabial flaps due to ORN in the maxillary region on the right side. Thirty-three patients were reconstructed with microvascular anastomosed flaps. These were fasciocutaneous flaps (e.g., radialis flaps) in 15 cases (12.7%), myocutaneous flaps (pectoralis and latissimus dorsi flaps) in two cases (1.7%), and osseous or osteo(myo)cutaneous flaps (scapula, fibula, and iliac crest flaps) in 16 cases (13.6%).

RT was performed in 95 patients (80.5%), with 11 patients receiving primary curative irradiation and 84 patients receiving adjuvant irradiation (initial irradiation dose: 64.6 ± 6.1 Gy). Additional chemotherapy was used in 47 patients (39.8%), mainly for advanced International Union Against Cancer (UICC) stage III and IV tumors.

### 3.2. Survival of Teeth and Implants after TT

After completion of TT, 1262 teeth were found in 87 patients. Twenty-three dentate patients had not been irradiated (212 teeth). At the time of the study, a total of 343 teeth (27.2%) had to be extracted.

For the analysis of implant survival, no implants were available that had already been placed prior to TT. Therefore, only the survival data of 328 implants placed in 71 patients after TT were considered. Implant placement was performed 50.6 ± 60.7 months following primary TT. Sixty-five implants were placed in patients who had not previously received RT. Definitive removable or fixed prostheses were placed at a mean of 8.9 ± 4.8 months after implant placement.

Of the 328 total implants placed, 23 (relative frequency 7.0%) were lost in 16 patients by the time of the study. These 23 implants had to be removed at a mean of 11.7 ± 14.9 months after implant placement. Early implant failure was found in 11 implants due to insufficient osseointegration. Late implant failure after functional loading was caused by peri-implantitis and, in two cases, the occurrence of ORN.

**Figure 1:**
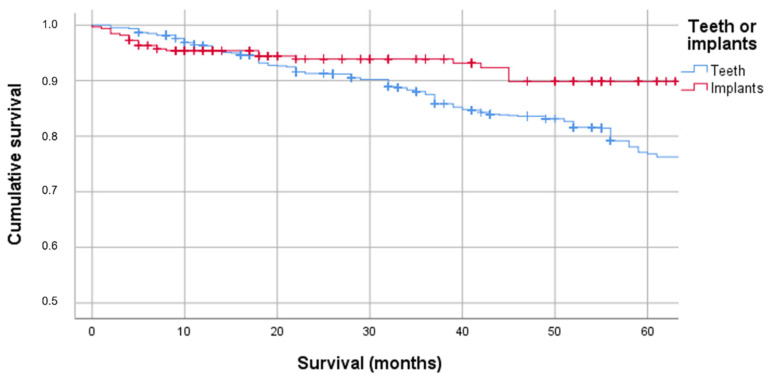
Cumulative survival of teeth and implants after tumor therapy.

The 5-year survival probability (5-YSP) of the teeth was 76.8% (95% CI: 74–79.6%), which was significantly lower than the 5-YSP of the implants (89.9%, 95% CI: 85.5–94.3%; *p* = 0.001; Figure 1).

### 3.3. Factors Influencing Implant Survival

RT produced no negative effect on implant survival. The 5-YSP of the implants of irradiated patients was higher than that of non-irradiated patients (86.2%; 95% CI: 75.1–97.3% vs. 90.8%; 95% CI: 86.0–95.6%; *p* = 0.103).

The implants in the mandible showed a higher 5-YSP of 91.5% (95% CI: 97.1–85.7%) than the implants in the maxilla (86.3%; 95% CI: 76.5–94.1%; *p* = 0.069).

Multivariable analysis of implant survival was performed using a marginal model. In the analysis, sex (4.1-fold increase in the risk of loss [RL] in women), chemotherapy (4.1-fold increase in the RL), and the presence of intraoral defects (9.1-fold increase in the RL) exhibited a pronounced influence on implant survival (Table 2). Regular nicotine abuse also increased the risk. The variables age, irradiation, assignment to the jaw, implant site, and SFR only slightly influenced implant survival. An increasing SFR was associated with a higher RL, in contrast to the tooth survival calculations.

## 4. Discussion

In the present study, differences were identified in the survival probabilities of teeth and implants after TT in the head and neck region. Thus, the null hypothesis, postulating that implants exhibit a higher probability of survival after TT than the patients’ own teeth, has to be proven.

We observed a relatively higher 5-YSP for the implants (89.9% [95% CI: 85.5–94.3%]) than for the teeth (76.8% [95% CI: 74–79.6%]). In the current literature, considerably higher 10-YSP of implants in non-irradiated patients are given (96.4% [95% CI 95.2%–97.5%]) [31]. Regarding these results, the implant survival rate of irradiated patients found in this study is to be assessed as considerably lower but nevertheless distinctly higher than the survival rate of irradiated patients’ teeth. Although the limitations of the study do not allow absolute conclusions to be drawn, it can be inferred from the results that implants may be recommended in patients after TT to the head and neck region. In our other publication, we analyzed the potential variables influencing the survival rate of teeth [16].

The frequency of loss of 7% observed for implants in the present study is in agreement with the findings reported in previous studies [32,33,34].

The probability of loss increased with increasing age. This correlation is already known for the patients’ own teeth [14]. Here, particularly in older adults compared to younger patients, a reduction in salivary flow, along with a reduction in both cognitive and manual skills, was found to complicate at-home oral hygiene and makes professional support essential [14,35,36]. These factors may exert greater influence in patients after TT and RT because the oral mucosa remains more sensitive years after radiation and anatomic changes further complicate the accessibility to implants.

Difficult accessibility not only causes difficulties for the patient in terms of oral hygiene at home but also makes implant insertion more difficult for the surgeon. This assumption is also supported by the regression results; patients with intraoral defects showed a significantly increased risk of implant loss. Besides a reduced ability to open the mouth, the jawbone is often anatomically altered with reduced height and width, requiring stronger implant inclinations or the insertion of implants with decreased diameters or length. These aspects represent worse conditions but are often accepted in this special patient group, as additional augmentative procedures in irradiated and already operated tissue involve additional risks. In addition to implant surface, implant length and diameter were already identified in previous studies as relevant factors influencing implant survival in patients without tumor history [9]. Due to the large number of variables already included in the analysis, these factors were not considered in this study. These aspects should be considered in the future, particularly in multicenter studies with a larger cohort.

RT exhibited no negative impact on implant survival, which was higher in irradiated patients than in non-irradiated patients but without statistical significance (90.8% vs. 86.2%; *p* = 0.103). A similar observation was described by Ettl et al. [37]. In a prospective clinical study, the authors evaluated the survival of Astra implants in patients after TT to the head and neck region and found a relative implant survival of 95.2% after one year. The authors distinguished implant placement in the non-irradiated jaw from that in the irradiated jaw within and outside the former target volume. They found the lowest survival rate after one year for implants in the former target volume (78.2%). The implants placed outside the target volume in the irradiated bone displayed higher survival rates (96.9%) than the implants in the non-irradiated bone (92.2%) [37]. The authors concluded that osseous healing was limited in the area of the former target volume, justifying the lower survival rates. They did not provide an explanation for the better survival rate of the implants in the irradiated bone outside the target volume compared to the implants in the non-irradiated bone. Currently, several factors can be hypothesized as causative. From a subjective clinical view, reduced bone volume, possibly with tongue adhesion and/or a deformed vestibule which makes a conventional denture impossible, or pronounced xerostomia, possibly caused by medication, are usually indications for therapy in non-irradiated patients with TT to the head and neck region. It is not uncommon to attempt an implant placement in borderline cases in the interest of the patient. The group of lost maxillary implants included, for example, two patients who lost five implants in the maxilla within the first 6 months. In these cases, to avoid additional postoperative morbidity, extensive augmentation procedures were not performed, and implants were placed in the existing residual bone. These individual cases could also explain why sex emerged as an influencing factor in the present study. Currently, no consensus can be found in the literature regarding this issue. However, in this study, early implant failure was mostly found in irradiated patients. In these cases, insufficient osseointegration might be caused by fibrotic changes and decreased vascularization of the bone. Otherwise, ORN was responsible for late implant failure in two cases. Chronical peri-implantitis might have led to the penetration of pathogens into the pre-damaged bone consisting of vulnerable, atrophic-fibrous tissue [18].

For the maxilla, the negative influence of the primarily softer bone quality on implant survival is often discussed. In the present study, maxillary implants also exhibited a 2-fold higher RL compared to mandibular implants. This result is consistent with the studies of Ettl et al. [37] and Sammartino et al. [7]. However, this trend was not confirmed by Di Carlo et al. [38]. The author group retrospectively studied 84 implants and found an influence for the interval between implantation and the end of TT but not for the implanted jaw. This time interval should be 12 months [7]. In the present study, all but one of the implants were placed after one year of freedom from recurrence. In this respect, the recommendations of the literature were followed [1,7,38].

The influence of the implant site on implant survival has been controversially discussed in the literature. Ch’ng et al. [39] found a higher loss rate for implants placed in osseo(-myo)cutaneous flaps, mainly fibular flaps. In a meta-analysis by Shugaa-Addin et al. [15], consideration was given to whether the implants had been placed in osseous grafts or microvascularly anastomosed osseo(-myo)cutaneous flaps. Although implants placed into the bone-grafted sites were generally considered to have a poorer prognosis, differences were also found in the prognostically better assessed microvascular anastomosed osseous or osteo(-myo)cutaneous flaps depending on the flap type. In the present study, no relevant difference was found regarding the implant survival related to grafts. However, the analysis made no differentiation between the different graft types. Contrary to what is stated in the literature, no generally increased RL of implants placed in the grafted bone was observed. In this context, it is interesting to note that the presence of intraoral defects did have an influence on the risk of implant loss. A 9-fold higher RL was found for the implants placed in patients with intraoral defects than in patients without intraoral defects. The explanations for this are manifold. Due to the variety of anatomical changes and the comparatively small cohort, a dedicated subdivision of the defect situation in the regression analysis was not performed. From a clinical point of view, however, it is understandable that the anatomical changes (e.g., in the case of a defect in the jaw angle with consequent tongue adherence and restricted mouth opening) make both implant placement and follow-up more difficult. These patients usually develop peri-implantitis, which is difficult to manage even with regular professional support.

Regular nicotine consumption is commonly considered to increase the risk of implant loss even in non-irradiated patients [40,41]. Experimental studies have shown that nicotine promotes the production of inflammatory cytokines, such as interleukin 6 or tumor necrosis factor α, by osteoblasts. Similarly, smokers were also found to have increased levels of proinflammatory cytokines in the peri-implant sulcus fluid. It was therefore concluded that nicotine and the chemicals contained in tobacco smoke may induce oxidative stress in both peri-implant soft and hard tissues. This can lead to an exaggerated immune response and progressive bone or implant loss [40,41]. Doll et al. [33] reported lower implant loss in irradiated and nonsmoking patients than in smokers. Clinically, most patients suffering from late implant failure showed signs of peri-implantitis, and an increased risk was also found for smokers in this study; however, it was less predictive than, for example, the presence of intraoral defects.

With regard to the SFR, a negative effect was observed according to the hazard ratio, i.e., greater amounts of saliva increased the risk of implant loss. The question arises here as to whether this apparent correlation does not mask other causalities. For example, patients are provided with implants despite good salivary flow, even if pronounced anatomical changes or a maximally reduced bone volume is present. These two factors also represent unfavorable conditions independent of the actual SFR. In view of the cohort size and the resulting limited significance of the results, the significance of this observation must therefore also be rated as limited.

The same approach is applicable to simultaneous chemotherapy. Currently, not many studies are available that investigate the influence of chemotherapy on implant survival. Ch’ng et al. [39] and Kovács [42] reported no relevant influence. In this context, the question arises whether chemotherapy acts as a surrogate factor for a higher UICC stage and, therefore, for a comparatively larger number of more difficult and often complication-prone implant-prosthetic rehabilitations.

Finally, the study’s limitations must be discussed critically. First, the validity of the selected start and end points for implant survival analysis must be critically questioned. The success, survival, and failure of implants have been described and defined on the basis of various variables [8]. Many studies have defined the starting point as the beginning of functional loading. In our study, we defined the implant survival period from the time of implant placement until the removal of the implant.

As previously described, patients with post-TT in the head and neck region represent a very special patient population. Dental treatment measures should be seen as part of the supportive therapy and, therefore, should be subordinated to the overall prognosis and general condition of the patient. Despite all advances in oncology treatment, the survival rate of patients is sometimes less than 50% after 5 years. Thus, in this patient population, it seems reasonable to look for adapted evaluation standards for implant success.

Based on clinical experience, implant planning, and placement, as well as follow-up in tumor patients often cannot be performed with the precision and under the standardization that is required in current implant studies. Often, despite the fabrication of surgical guides and preoperative determination of implant length and diameter, operators intraoperatively deviate from the original planning due to clinical bone and/or soft-tissue conditions. After healing and implant uncovering, soft-tissue interventions often follow, especially in cases with tongue margin and oral floor defects. In these cases, uncovered implants serve as anchoring elements for the fixation of bandage or expansion plates (e.g., in case of scar traction in the area of the lips). These are yet to represent the definitive form of the prosthesis but still represent a load due to the anchoring on the implants. In some cases, temporary prostheses that support the surgical soft-tissue corrections are incorporated for quite a few months. Patients often perceive this simple, temporary solution as an enormous, subjective progress. Since it is difficult to decide in such cases when the implant can be considered as finally loaded, we decided to use the insertion time as the starting point for the survival analysis for all implants. Another aspect that was decisive in this context is the risk of ORN. Every implant placement carries the risk of bone infection, which means that every attempt at implant placement could be considered an additional risk factor for the development of ORN. This must, of course, be considered in the overall context of irradiated patients.

Due to the large number of variables included in the analysis, the comparatively small patient cohort, the varying observation periods, and the partially retrospective data collection on the basis of disease documentation, the results of the current study have to be interpreted critically. Future prospective multicenter studies with larger patient cohorts are necessary to validate the conclusions presented.

## 5. Conclusions

Due to the comparatively low number of patients, varying findings, and fluctuating observation periods, the following tendencies can be highlighted.

Implants have a higher survival probability than teeth after TT. In patients who experience progressive tooth decay within a short time after the end of TT despite regular conservative follow-up, implant-supported restorations should be considered in preference to tooth-supported restorations.

## Figures and Tables

**Table 1:** Tumor localization and tumor stage according to ICD-10 and UICC.

Localization	ICD-10	UICC-Stadium							Number (%)
		I	II	III	IV A	IV B	IV C	n.d.	
Nasopharynx	C11, C30, and C31	0	1	0	2	2	0	2	7 (5.9)
Tonsil	C09 and C10	0	2	1	6	4	0	1	14 (11.9)
Tongue base	C01	0	2	1	4	0	0	1	8 (6.8)
Oral cavity	C00, C02-C06, and C08	9	13	11	12	6	1	5	57 (48.3)
Cheek/parotid gland	C06 and C07	3	1	3	1	0	0	0	8 (6.8)
Larynx/hypopharynx	C12, C13, and C32	0	5	4	7	0	0	0	16 (13.6)
Others	C41, C49, C80, and D18	1	0	0	2	2	0	3	8 (6.8)
Number (percent)		13 (11.0)	23 (19.5)	21 (17.8)	34 (28.8)	14 (11.9)	1 (0.8)	12 (10.2)	118 (100)

ICD, International Statistical Classification of Diseases and Related Health Problems; UICC, International Union Against Cancer; n.d., not defined.

**Table 2:** Hazard ratios of demographic and clinical characteristics for multivariable analysis of implant survival.

Variable	Reference	Hazard Ratio	95% CI		*p*-Value
Age	-	1.024	0.970	1.081	0.384
Sex	Male	4.106	1.357	12.423	0.012
SFR	-	1.637	0.706	3.797	0.251
RT	No RT	0.347	0.094	1.282	0.113
Nicotine abuse	No nicotine abuse	2.670	0.847	8.417	0.094
Implant position	Mandible	1.830	0.816	4.103	0.143
Intraoral defect	No intraoral defect	9.117	0.878	94.720	0.064
Implant site	Autogenous bone	0.367	0.078	1.738	0.207
Chemotherapy	No chemotherapy	4.127	1.240	13.738	0.021

SFR, salivary flow rate; RT, radiotherapy; CI, confidence interval.

## Data Availability

The data presented in this study are available on request from the corresponding author due to privacy restrictions.
